# Towards ultrasound‐guided adaptive radiotherapy for cervical cancer: Evaluation of Elekta's semiautomated uterine segmentation method on 3D ultrasound images

**DOI:** 10.1002/mp.12325

**Published:** 2017-06-16

**Authors:** Sarah A. Mason, Tuathan P. O’Shea, Ingrid M. White, Susan Lalondrelle, Kate Downey, Mariwan Baker, Claus F. Behrens, Jeffrey C. Bamber, Emma J. Harris

**Affiliations:** ^1^ Joint Department of Physics at the Institute of Cancer Research and Royal Marsden NHS Foundation Trust Sutton and London UK; ^2^ Department of Oncology Herlev Hospital, University of Copenhagen Herlev Denmark

**Keywords:** adaptive radiotherapy, cervical cancer, segmentation, ultrasound, uterus

## Abstract

**Purpose:**

3D ultrasound (US) images of the uterus may be used to adapt radiotherapy (RT) for cervical cancer patients based on changes in daily anatomy. This requires accurate on‐line segmentation of the uterus. The aim of this work was to assess the accuracy of Elekta's “Assisted Gyne Segmentation” (AGS) algorithm in semi‐automatically segmenting the uterus on 3D transabdominal ultrasound images by comparison with manual contours.

**Materials & methods:**

Nine patients receiving RT for cervical cancer were imaged with the 3D Clarity^®^ transabdominal probe at RT planning, and 1 to 7 times during treatment. Image quality was rated from unusable (0)–excellent (3). Four experts segmented the uterus (defined as the uterine body and cervix) manually and using AGS on images with a ranking > 0. Pairwise analysis between manual contours was evaluated to determine interobserver variability. The accuracy of the AGS method was assessed by measuring its agreement with manual contours via pairwise analysis.

**Results:**

35/44 images acquired (79.5%) received a ranking > 0. For the manual contour variation, the median [interquartile range (IQR)] distance between centroids (DC) was 5.41 [5.0] mm, the Dice similarity coefficient (DSC) was 0.78 [0.11], the mean surface‐to‐surface distance (MSSD) was 3.20 [1.8] mm, and the uniform margin of 95% (UM95) was 4.04 [5.8] mm. There was no correlation between image quality and manual contour agreement. AGS failed to give a result in 19.3% of cases. For the remaining cases, the level of agreement between AGS contours and manual contours depended on image quality. There were no significant differences between the AGS segmentations and the manual segmentations on the images that received a quality rating of 3. However, the AGS algorithm had significantly worse agreement with manual contours on images with quality ratings of 1 and 2 compared with the corresponding interobserver manual variation. The overall median [IQR] DC, DSC, MSSD, and UM95 between AGS and manual contours was 5.48 [5.45] mm, 0.77 [0.14], 3.62 [2.7] mm, and 5.19 [8.1] mm, respectively.

**Conclusions:**

The AGS tool was able to represent uterine shape of cervical cancer patients in agreement with manual contouring in cases where the image quality was excellent, but not in cases where image quality was degraded by common artifacts such as shadowing and signal attenuation. The AGS tool should be used with caution for adaptive RT purposes, as it is not reliable in accurately segmenting the uterus on ‘good’ or ‘poor’ quality images. The interobserver agreement between manual contours of the uterus drawn on 3D US was consistent with results of similar studies performed on CT and MRI images.

## 1 Introduction

Uterine motion reduces the accuracy of external beam radiotherapy (RT) for cervical cancer,^1^, ^2^ with positional changes ranging from 2 to 60 mm between treatments.^2^, ^3^, ^4^, ^5^ To compensate for this positional uncertainty of the uterus, the planning target volume (PTV) for the primary tumor site (i.e., excluding nodal disease) is commonly generated by expanding the clinical target volume (CTV) by 6–40 mm.^6^ This leads to increased dose to surrounding normal tissues and incidence of adverse effects (such as both chronic and acute bladder, gastrointestinal, and hematological toxicities) and in addition, may not be sufficient for adequate uterus coverage in some cases.^2^, ^7^, ^8^, ^9^, ^10^, ^11^.

At present, most verification schedules rely on either megavoltage portal imaging or cone beam CT (CBCT) imaging of the bony anatomy. These images are commonly reviewed immediately prior to radiation delivery, and are used to correct for random errors by shifting the couch to align the patient's bony anatomy position during treatment with its position during planning (i.e., position in the CT simulation [SIM] image).^12^ However, a perfect bone‐match does not guarantee correspondence between the soft‐tissues; residual uncertainty regarding the shape and position of the uterus remains.^1^, ^2^ One approach to correct for this uncertainty uses fiducial markers as a surrogate for soft‐tissue imaging. Markers can be inserted into the uterus and imaged with x‐ray‐based modalities, though this is invasive and not always reliable as the fiducials can migrate.^6^, ^13^, ^14^

The Clarity^®^ ultrasound‐guided RT (USGRT) system (Elekta Ltd., Stockholm, Sweden) has been developed to provide soft‐tissue imaging to improve the accuracy of RT for gynecological cancer compared with bony anatomy‐based image guidance. Briefly, the Clarity^®^ system may be used to acquire ultrasound images in the planning CT room (US‐SIM) and treatment room (US‐Tx) frame of reference using an infrared‐tracked transducer that is spatially calibrated to the treatment co‐ordinate system.^15^ In the context of cervical cancer RT, this technology allows the user to localize the uterus on US with respect to the isocenter of the RT treatment room. This could enable: (a) soft‐tissue‐based couch shifts and/or (b) adaptive RT, where the uterine shape at the time of treatment is explicitly taken into account. Although soft‐tissue‐based couch shifts resulting from USGRT may improve the alignment of the uterine centroid with the treatment room isocenter, they do not address the issue of healthy‐tissue sparing because large margins to account for organ deformation are still required. Adaptive RT is therefore an attractive alternative because the RT beam aperture can be modified according to the shape and position of the target at the time of RT delivery to ensure adequate target coverage while minimizing the organ at risk (OAR) radiation exposure. Segmentation of the uterus could allow for automated selection of the plan‐of‐the‐day from a library of predefined treatment plans, or for online treatment replanning according to the patient's anatomy at each treatment fraction.^5^, ^16^, ^17^.

Manual contouring by an expert can be considered a gold standard for organ segmentation, though this is too time consuming to be a feasible option for online adaptive RT.^18^, ^19^ Online segmentation must be achieved on a timescale of minutes so that the additional time that the patient spends on the treatment couch during segmentation does not result in patient discomfort and/or movement, a delay in the clinical workflow, or significant natural changes in internal anatomy (such as bladder filling) that would displace the uterus from its position when it was first imaged. For such applications, a rapid method of capturing the 3D uterine outline at treatment time is greatly needed.

**Figure 1:**
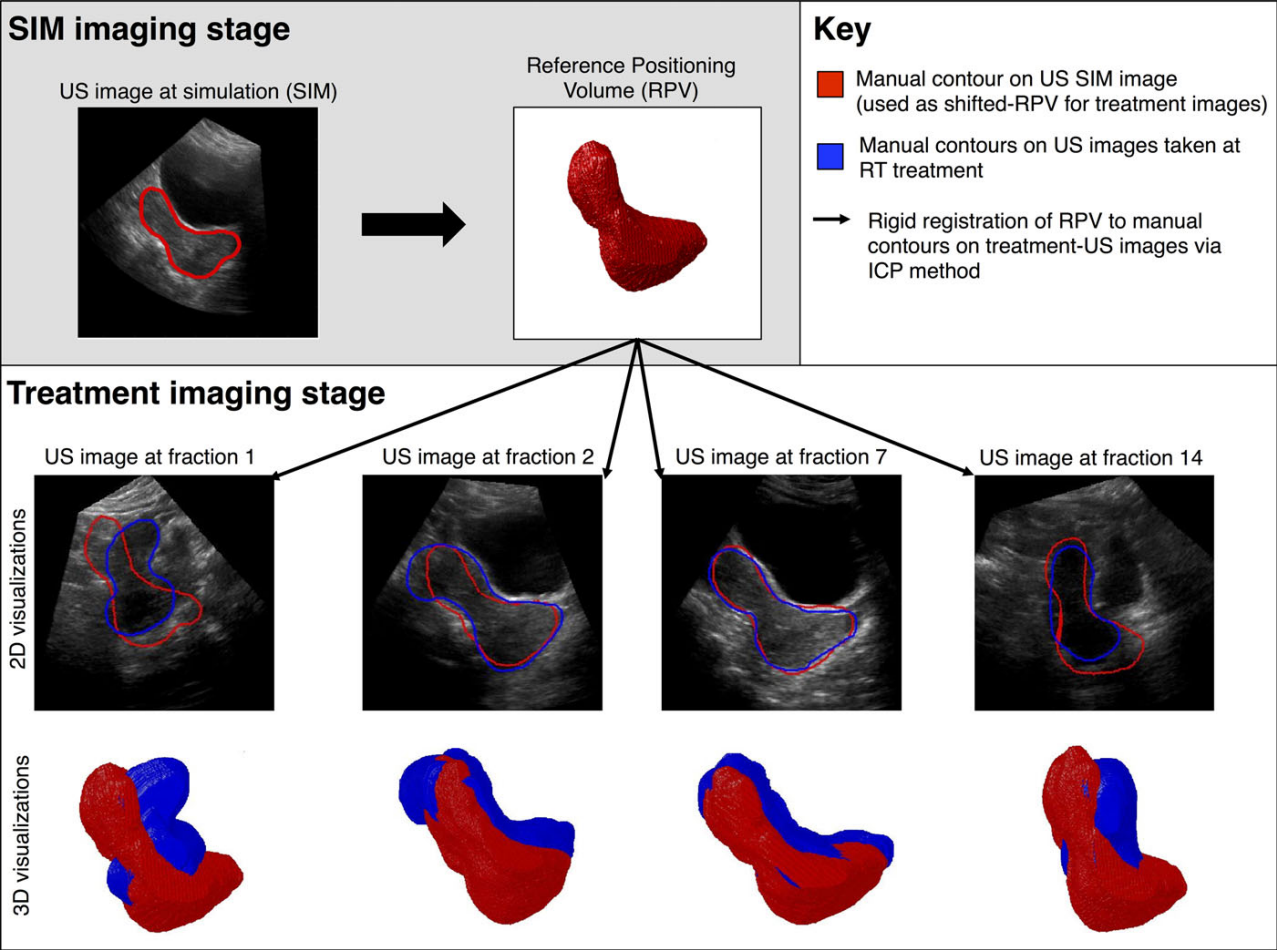
Example of rigid registration ROI localization technique, where a Reference Positional Volume (RPV) from SIM is used to localize the uterus as visualized by US at treatment (US‐Tx). Note that the RPV is often a poor representation of the uterus at the time of treatment (particularly at US‐Tx1 and US‐Tx14) due to the large amount of uterine deformation. [Color figure can be viewed at wileyonlinelibrary.com]

One method of localizing regions of interest (ROIs) at treatment is to incorporate *a priori* knowledge of ROI shape and size, which can be obtained from US‐SIM. The Clarity^®^ system implements this approach by requiring a user to manually shift a Reference Positional Volume ([RPV] — the set of rigid manual ROI contours drawn on the US‐SIM image) to best match the apparent position of the ROI as visualized by US‐Tx. This allows for estimation of the ROI centroid position for soft‐tissue‐based patient setup. However, in the context of adaptive RT, this approach requires that the ROI undergo little or no deformation throughout the course of treatment so that the RPV is still a valid representation of the patient's anatomy at the time of radiotherapy delivery. As the large amount of deformation occurring in the uterus violates this constraint, rigid registration‐based techniques (including Clarity's^®^ RPV method) for localizing the uterus at the time of treatment are not suitable for adaptive radiotherapy, as shown in Fig. 1.^28^

An alternative to manual contouring is to use a segmentation algorithm to automatically or semiautomatically (i.e., where user‐interaction is required) contour the uterus in 3D in place of an expert. To our knowledge, Elekta is the first to develop an automated solution for segmenting the uterus on 3D transabdominal US images via the “Assisted Gyne Segmentation” (AGS) tool.^20^ However, similar to the RPV method, the AGS tool is currently only used to guide soft‐tissue‐based couch shifts according to the apparent centroid position at treatment.

There may be considerable patient benefit in adaptive RT from employing a method that can automatically, and hence rapidly, segment the 3D uterine shape on 3D US images. However, neither the AGS tool nor any other method for automatically segmenting the uterus has yet been assessed for its accuracy and hence potential for application in adaptive RT. In this work, the following research questions were addressed:

1. What is the accuracy of the AGS tool in segmenting the uterus on 3D transabdominal US images? This was quantified by pairwise comparison with corresponding manual contours, which led to the secondary research question.
2. What is the interobserver variability in contouring the uterus on 3D transabdominal US images? This variability was used as a reference for the ideal accuracy of a semiautomated segmentation method.
3. What is the effect of image quality on both (a) AGS tool accuracy and (b) interobserver contour variation.

All analyses were performed on 3D transabdominal US images acquired from nine cervical cancer patients.

## 2 Materials & methods

### 2.A. Data acquisition

Nine patients receiving radiotherapy for cervical cancer were included in this study: six from Herlev Hospital, Copenhagen, Denmark (23 US images acquired) and three from the Royal Marsden NHS Foundation Trust, London, UK (21 US images acquired). Ethics approval for these studies was obtained from the ‘De Videnskabsetiske Komiteer’ and the ‘NHS Research Ethics Committees (reference: 15/LO/1438)’, respectively. Median patient age was 49.5 yr (range 36–65 yr), median body mass index (BMI) was 27.6 (range 21.5–40.7), and median FIGO cervical cancer stage was IIB (range IIB–IIIB). The six patients from Herlev were instructed not to pass urine approximately 1 hour prior to RT treatment. The three patients from the Royal Marsden Hospital were asked to drink 200 mL of liquid and to refrain from passing urine in the hour prior to treatment. After being positioned on the couch, 3D transabdominal US images of the uterus were acquired for each patient at 2 to 8 times (once at US‐SIM and 1‐7 times at US‐Tx) during the course of treatment. All scans were acquired with the Clarity^®^ USGRT system (Clarity^®^ Model 310C00, Elekta, Montreal, Canada), using a 3D mechanically swept convex 5 MHz transducer (m4DC7‐3/40), with the pressure between the US transducer and the patient's skin as low as possible to minimize soft‐tissue displacement.

### 2.B. Segmentation

*Manual Segmentation:* Four experts [two clinical oncologists (IMW and SL), one radiologist (KD), and one researcher trained by an oncologist (SAM)] manually contoured the uterus in the sagittal plane on a RayStation 5.0 workstation (RaySearch Laboratories, Stockholm, Sweden) for all US‐SIM and US‐Tx images analyzed. In this study, the ‘uterus’ is referred to as a single structure containing both the uterine body and cervix.

**Figure 2:**
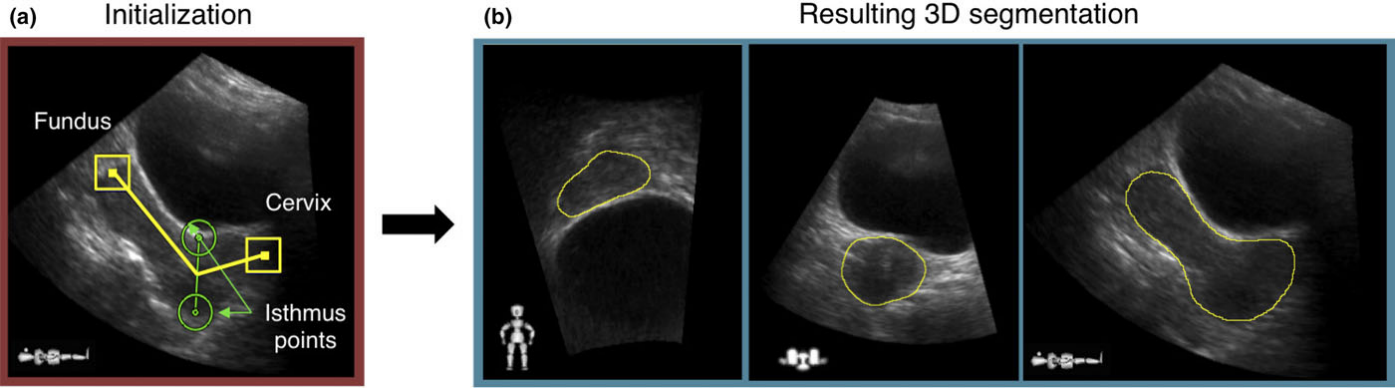
(a) Snapshot of AGS tool user interface and (b) resulting segmentation, where the placement of four anatomical hint points is required to initialize AGS segmentation. The hint points are the uterine fundus and base of the cervix (squares), and the anterior and posterior isthmus points (circles). The resulting segmentation is shown in on three slices from the coronal, transversal, and sagittal planes, respectively. [Color figure can be viewed at wileyonlinelibrary.com]

*AGS segmentation:* The core of the AGS tool is a discrete dynamic contouring (DDC) algorithm, which is a gradient‐based segmentation technique commonly used in prostate segmentation applications.^22^ Elekta have adapted the methods employed by Ladak et al.,^18^ Hu et al.,^23^ and Ghanei et al.,^24^ such that the algorithm semiautomatically segmented the uterus on US. The same four experts who performed the manual uterine segmentations used the AGS tool to segment the uterus on all US image volumes. This required an initialization step where four hint points were placed on uterine features (the uterine fundus, both isthmus points, and base of the cervix) on a central sagittal slice (Fig. 2).

### 2.C. Data analysis

#### 2.C.1. Image quality rating

Each 3D US image was rated twice on a 4‐point scale according to the criteria listed in Table 1 by one observer (SAM), with at least 10 days in between ratings of the same image. Any image receiving a rating of 0 at least once was excluded from further analysis. The final rating for the remaining images was calculated as the mean rating for each image, rounded to the nearest integer.

**Table 1:** Scale used to rate image quality of transabdominal US uterine scans

Rating	Criteria
0 ‐ Unusable	Impossible to identify any structures in the US image
1 ‐ Poor	Uterine boundaries blurred in the majority of planes and slices. Imaging artifacts severe and/or prevalent
2 ‐ Good	Uterine boundaries may be partially obscured or difficult to discern due to moderate imaging artifacts, but still visible in the majority of slices and anatomical planes
3 ‐ Excellent	Clearly defined uterine boundaries in all three anatomical planes. Subtle or no imaging artifacts present

#### 2.C.2. Contour agreement

Interobserver manual contouring variation was assessed by measuring the pairwise agreement between the four manual contours drawn on each US image; i.e., each observer contour was compared with the other three observers’ contours giving 12 pairwise comparisons per image. The accuracy of the AGS tool was quantified by measuring its agreement with manual contours via pairwise analysis; i.e., each AGS contour was compared with each manual contour, giving 16 pairwise comparisons per image. In some instances, the AGS algorithm did not produce a contour at all; these cases were referred to as failed segmentation attempts, and were excluded from the quantitative analyses. The AGS segmentation attempts that failed were reported as a percentage of all AGS segmentations attempted. In all cases, ‘contour agreement’ was assessed using the following four metrics, where **A** and **B** represent hypothetical 3D contours:

1. The Euclidian distance between the centroids (DC) of **A** and **B**. The centroid of the uterus (a point identified by its x, y, and z coordinates in the treatment room frame of reference) is currently used in the Clarity${}^{®}$ workflow to suggest soft‐tissue‐based couch shifts; discrepancies between **A** and **B** were considered to be setup errors in the patient position. A perfect DC was defined as 0 mm.
2. The 3D Dice similarity coefficient (DSC), defined as (2|**A**∩**B**|)/(|**A**|+|**B**|), where a DSC of 0 and a DSC of 1 indicate zero and perfect overlap, respectively. Good agreement (across a range of anatomical sites and imaging modalities) was considered to be ∼ > 0.75.^25^, ^26^, ^27^
3. The mean surface‐to‐surface distance (MSSD) was defined as the mean of the Euclidean distances between every vertex on the surface of **A** and its nearest neighboring vertex on the surface of **B**. Like the DSC, the MSSD is a measure of segmentation accuracy, though it is more sensitive to strong local deviations in shape. A perfect MSSD was defined as 0 mm, and good contour agreement (across a range of anatomical sites and imaging modalities) was considered to have an MSSD of ∼ 3 mm or less.^28^, ^29^, ^30^, ^31^
4. The Uniform Margin of 95% (UM95)^28^ was defined as the margin required (in mm) to uniformly expand **A** to create **A’**, such that at least 95% of the volume of **B** was included in the volume of **A’**. The UM95 was used to indicate the contribution of localization accuracy to the overall treatment margin required in RT.

#### 2.C.3. Statistical analyses

*Interobserver manual contour agreement:* A Wilcoxon rank sum test with Bonferroni correction was used to test for differences in DC, DSC, MSSD, and UM95 between manual contours in each image quality rating group (1, 2, and 3) to see whether agreement between observers increased with improving image quality.

*AGS segmentation accuracy:* A Wilcoxon rank sum test was used to test for differences in DC, DSC, MSSD, and UM95 between AGS and manual contours for all images, and when the images where grouped according to image quality (ratings 1, 2, and 3). The interobserver manual contour agreement was used as a benchmark to gauge the performance of semiautomatic segmentation methods; ideally, the agreement between an algorithmically derived contour and a manually derived contour should be the same as the variation in agreement between manual contours. To investigate whether better image quality improved AGS segmentation performance, a Wilcoxon rank sum test with Bonferroni correction was used to test for differences in DC, DSC, MSSD, and UM95 within each group (image quality ratings of 1, 2, and 3).

## 3 Results

**Figure 3:**
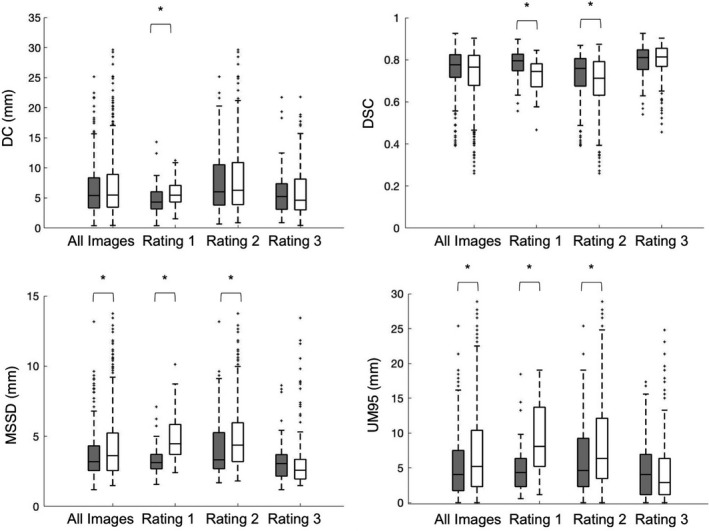
Boxplot showing interobserver variability between manual contours (shaded boxes) and the accuracy of the AGS algorithm as measured by agreement with manual contours (white boxes). The asterisks denote statistical differences between manual and AGS segmentations (*P* < 0.05). Note that there were no significant differences between the AGS and manual segmentations in images with a quality rating of 3 (excellent) on any metric considered. Also note the that the AGS segmentations were significantly different from manual contours on rating 1 (poor) quality images for every metric considered. *Abbreviations*: DC = distance between centroids, DSC = Dice similarity coefficient, MSSD = mean surface‐to‐surface distance, and UM95 = uniform margin of 95%.

*Image quality rating*: 35 of the 44 US images acquired had an image quality rating of 1 or higher, and were included in subsequent quantitative analyses: 6/35, 18/35, and 11/35 US images received ratings of 1, 2, and 3, respectively. *Interobserver manual contour agreement*: The median [interquartile range (IQR)] DC, DSC, MSSD, and UM95 results for the interobserver manual contouring variation are given in Table 2, and Fig. 3. The overall medians [IQR] for the DC, DSC, MSSD, and UM95 were 5.41 [5.0] mm, 0.78 [0.11], 3.20 [1.8] mm, and 4.04 [5.8] mm, respectively. Images with a quality rating of 2 had a significantly lower (*P* < 0.05) DC, DSC, and MSSD than images with a quality ratings of 1 or 3 in every metric but UM95 (Table 2). There was no statistical difference between images with a ranking of 1 and 3 in any of the agreement metrics considered.

**Table 2:** *Col 3*: Agreement (median [IQR]) between manual contours from different observers and *Col 4*: accuracy of the AGS tool, measured by pairwise analysis with manual contours. Symbols indicate statistical differences (*P* < 0.05) between image ratings within a particular group: *γ* ‐ statistically different to Rating 1, *δ* ‐ statistically different to Rating 2, and *ϕ* ‐ statistically different to Rating 3. Note that the AGS tool accuracy was significantly better in Rating 3 images than Rating 2 images (all cases) and rating 1 images (DSC, MSSD, UM95). (*Abbreviations*: DC = distance between centroids, DSC = Dice similarity coefficient, MSSD = mean surface‐to‐surface distance and UM95 = uniform margin of 95%.)

	Image quality	Interobserver variability (manual contours)	AGS tool accuracy
DC(mm)	**All Images**	**5.41[5.0]**	**5.48[5.45]**
	Rating 1	4.33 [2.9]	5.46 [2.8]
	Rating 2	6.03 [6.7]${}^{\gamma ,\phi }$	6.29 [7.0]
	Rating 3	5.26 [4.3]	4.64 [5.12]${}^{\delta }$
DSC	**All Images**	**0.78 [0.11]**	**0.77 [0.14]**
	Rating 1	0.79 [0.08]	0.74 [0.11]
	Rating 2	0.76 [0.13]${}^{\gamma ,\phi }$	0.71 [0.16]
	Rating 3	0.81 [0.09]	0.81 [0.09]${}^{\gamma ,\delta }$
MSSD (mm)	**All Images**	**3.20 [1.8]**	**3.62 [2.7]**
	Rating 1	3.13 [0.08]	4.47 [2.1]
	Rating 2	3.33 [1.0]${}^{\phi }$	4.38 [2.8]
	Rating 3	3.06 [1.5]	2.58 [1.4]${}^{\gamma ,\delta }$
UM95 (mm)	**All Images**	**4.40 [5.8]**	**5.19 [8.1]**
	Rating 1	4.33 [4.0]	8.08 [8.5]
	Rating 2	4.62 [6.9]	6.35[8.7]
	Rating 3	4.01 [5.8]	2.89 [5.2]${}^{\gamma ,\delta }$

*AGS contours acquired*: Out of 140 attempts at using the AGS tool to segment the uterus (35 US images * 4 observers), 113 AGS contours were successfully obtained (80.7%), whereas the algorithm failed to return a result in 27 cases (19.3%). The 27 cases with no result were excluded from the quantitative analysis.

*AGS segmentation accuracy*: The median [IQR] DC, DSC, MSSD, and UM95 results for the AGS segmentation accuracy are given in Table 2. The AGS segmentations had a significantly better accuracy (i.e., agreement with manual contours) on images with a rating of 3 than images rated 1 or 2. However, there was no difference in segmentation performance between rating 1 images and rating 2 images. The AGS algorithm agreed with manual contours on images that received a rating of 3, as there was no significant difference between them in all metrics considered (Fig. 3). However, the AGS algorithm was less accurate in segmenting the uterus on rating 1 images according to all metrics considered, and also less accurate on rating 2 images according to DSC, MSSD, and UM95. Overall, the AGS algorithm was statistically equivalent to manual contouring in terms of DC and DSC, but not in terms of MSSD and UM95.

## 4 Discussion

*Image quality rating*: Low bladder volume and high BMI can increase the attenuation of US and reduce image quality.^32^, ^33^ Not only does a full bladder help with tissue sparing in RT treatment for cervical cancer but it also has the added benefit of providing an acoustic “window" to the uterus, as urine has a low US attenuation coefficient compared with surrounding tissues. Patients with a high BMI are likely to have a greater amount of adipose tissue through which the US must travel, which may be important because fat has a relatively low speed of sound and its presence can cause image aberrations due to acoustic refraction, wave aberration, reverberations, steering errors, focusing errors, and spatially dependent image scale miscalibration. These factors may explain why eight of the nine of the unusable images (i.e., received an image rating of ‘0’) were acquired from patients who did not follow a stringent drinking protocol (the Herlev cohort), and why four of the nine unusable images were obtained from the same patient, who had the highest BMI (36.5) of the patients included in this study. Additionally, care was taken to apply low pressure to the abdomen when acquiring the US images to avoid internal soft‐tissue displacement; though this is crucial for RT applications, this comes at the cost of poorer image quality as contact between the transducer and the skin surface is decreased.^34^, ^35^ A larger study is needed to investigate methods of overcoming these challenges associated with implementing US guidance in adaptive RT to reduce the risk of obtaining an unusable image. One potential solution could be to ensure an adequate level of bladder filling at the time of treatment by enforcing a stringent drinking protocol, or by finding ways to compensate for variables such as poor hydration over the previous twenty four hours prior to treatment or reduced bladder capacity often occurring during treatment. Another solution could be establishing inclusion/exclusion criteria to identify good candidates for transabdominal US scanning. However, it should be noted that even without such measures in place, approximately 80% of the US images acquired in this study were used to successfully identify the position and shape of the uterus at the time of RT treatment.

*Interobserver manual contour agreement*: The DC, DSC, and MSSD values reported here (medians of 5.4 mm, 0.78, and 3.20 mm, respectively) are consistent with those reported in similar studies, though a direct comparison was not possible due to differences in: imaging modalities used, the disease status of the cohort investigated, the anatomical site contoured, and the number of observers. Baker et al. reported a median DC of 6.0 mm between contours of two observers in manually delineating the uterus on 3D US on a healthy volunteer cohort.^36^ In the literature, reported values of the DSC between manual contours drawn on CT and MRI images for a variety of anatomical sites ranged from ∼ 0.7–0.98^25^, ^26^, ^37^, ^38^ with ∼ 0.7–0.8 generally considered acceptable.^25^, ^26^, ^27^ The MSSD between manual contours drawn on US, CT, and MRI images reported in the literature for a variety of anatomical sites ranged from ∼ 1–5 mm.^26^, ^31^, ^39^, ^40^ The fact that the UM95 required to overcome interobserver contouring variability in this study (median [IQR] of 4.04 [5.8] mm) was much smaller than the interfractional uterine motion commonly observed, (which can be as much as 60 mm) supports the idea that USGRT could reduce the size of the margins needed to compensate for organ motion, even in the presence of contouring uncertainties.^5^

**Figure 4:**
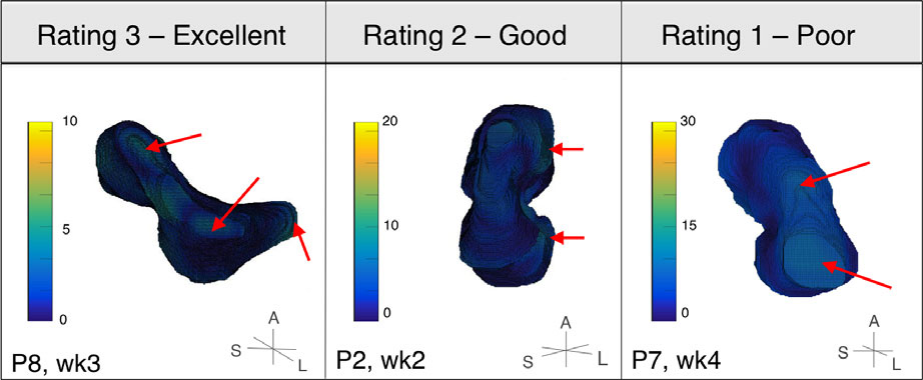
Heatmaps showing interobserver manual contour variability displayed on the uterine isosurface. Dark blue (black in print) indicates 0 mm deviation, and yellow (white in print) indicates > 10 mm deviation. The scale of the heatmap is different for each image. Note that the largest deviations tend to be in the left–right uterine edges and the base of the cervix (red arrows, black in print). The orientation of the uterus is given by axes in bottom right corner (A = anterior, S = superior, and L = left) Labels in bottom left corner of each image indicate the patient number (P2, P7, or P8) and time point where image was obtained (week = wk). For corresponding US image, see row 1 of Fig. 5. [Color figure can be viewed at wileyonlinelibrary.com]

As shown in Fig. 4, common areas of disagreement between manual contours observed in this study arose from determining the left–right extent of the uterus, and distinguishing the base of the cervix from the top of the vagina. This may be attributed to problems associated with contouring in the sagittal plane. The agreement between manual contours did not correlate with improving image quality, despite the uterine boundary becoming sharper in higher quality images. This may be due to the experts’ abilities to infer the boundary of the uterus in places where it was obscured using prior knowledge of uterine shape and/or relative orientation of other anatomical landmarks in the US field of view. Even in the presence of these sources of disagreement, the manual contour agreement reported here is comparable with previous contouring variability studies, indicating that the uterus can be visualized with 3D transabdominal US at the time of RT treatment. Furthermore, USGRT could be dosimetrically beneficial to cervical cancer patients as the component of the margin needed to compensate for contouring variability (represented by the UM95) is still much smaller than the margin that is needed to compensate for uterine motion without any form of soft‐tissue guidance.

*AGS tool performance*: When applied to images acquired from cervical cancer patients at RT treatment, the AGS tool failed to return a result in nearly 20% of segmentation attempts, which is unacceptable for use in adaptive RT considering that an ideal segmentation method should produce a result in 100% of segmentation attempts. This occurred in cases where the image quality rating was 2 or lower, indicating that a clearly defined boundary in all three anatomical planes is required to ensure that the AGS tool functions. Potential solutions for improving the image quality such that the probability of AGS returning a result is increased may include introducing a selection criteria at baseline to identify patients who have characteristics conducive to obtaining excellent US images (e.g., low BMI), or applying US image processing/acquisition techniques such as speckle reduction or image compounding to improve the contrast to noise ratio between the uterus and background tissues.^42^, ^43^, ^44^

**Figure 5:**
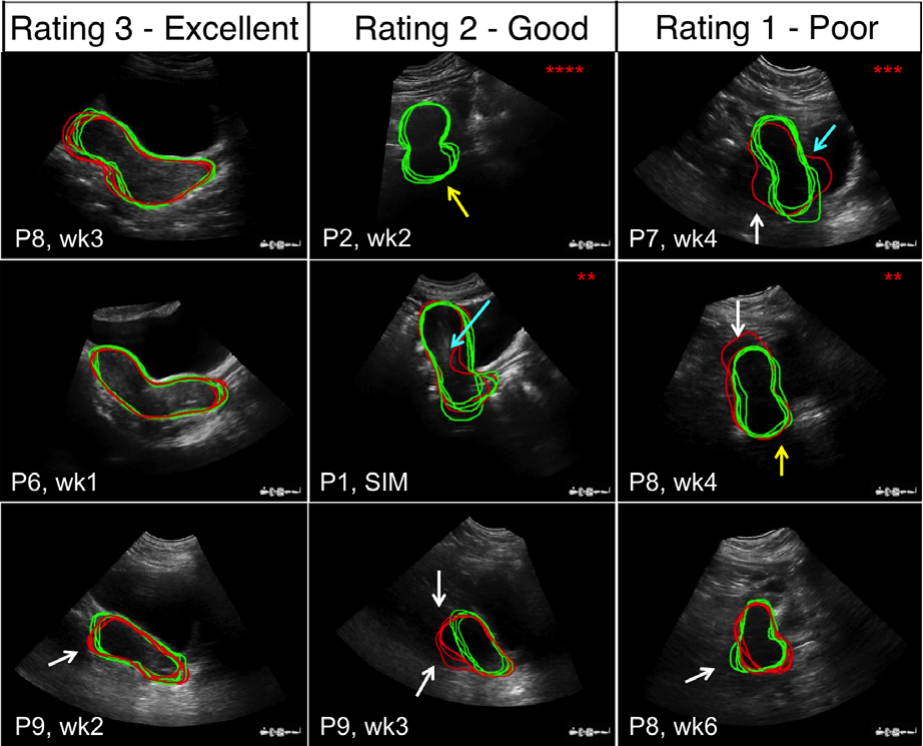
Examples of manual (green online, light gray in print) and AGS (red online, dark gray in print) contours superimposed over a central slice of corresponding 3D US images. Images are grouped by column according to image quality rating. Labels in bottom left corner indicate the patient number (P1‐P9) and time point where image was obtained (week = wk and SIM = ultrasound acquired at CT simulation). Arrows indicate artifacts in the US image that have led to errors in the AGS algorithm. White arrows = shadowing, yellow (white in print) arrows = signal attenuation, and cyan (gray in print) arrows = misinterpretation of other anatomical boundaries as the uterine boundary (e.g., the endometrium in P1 SIM and the bladder in P7 week 4). Asterisks indicate number of times where the AGS algorithm failed to give a result for the corresponding image. [Color figure can be viewed at wileyonlinelibrary.com]

In the 80% of cases where a result was returned, the values of DC, DSC, and MSSD between AGS and manual contours were dependent on image quality. The agreement between the AGS algorithm and manual contours was statistically equivalent to the interobserver agreement between manual contours for images with a rating of 3; this indicates that the AGS algorithm can accurately segment the uterus on US images containing virtually no imaging artifacts/imperfections. This is shown in column 1 of Fig. 5, where the AGS (red) segmentations agree well with the manual (green) segmentations in on the US images with distinct, continuous uterine boundaries. Note that in these cases, the patients all had full bladders extending across the length of the uterus. However, the majority of the US images acquired in this study had some form of image artifact partially obscuring the true uterine boundary (image quality ratings 1 and 2). In these cases, the AGS algorithm performance was significantly poorer than its manual segmentation counterpart on all metrics considered (with the exception of the DC on rating 2 images), which may be attributed to the fact gradient‐based algorithms are susceptible to errors due to the speckle, shadowing, and signal variation with ultrasound beam angle commonly present in US images taken of cervical cancer patients during RT treatment,^18^ as shown in columns 2 and 3 of Fig. 5. In these examples, the image artifacts either caused the AGS contour to deviate from the true uterine boundary (as defined by the manual segmentations), or confounded the US image to extend that the resulting shape of the uterus defined by the AGS tool was either corrupted, or unobtainable, despite good agreement between the corresponding manual contours. Furthermore, the statistical analyses performed to check for differences in AGS algorithm accuracy between image rating groups showed that AGS segmentations on images with a rating of 3 were significantly better than AGS segmentations on images with ratings 1 or 2.

When comparing the overall performance of the AGS algorithm with the interobserver manual contours, there were significant differences in MSSD and UM95, but no significant differences in DC or DSC. Note that (a) DC does not take shape into account and (b) the DSC is only sensitive to changes in shape if that shape is accompanied by changes in the volume of overlap; for example, thin extrusions of the contour produced by the AGS algorithm in the presence of shadowing or speckle had little effect on the DSC, (c) the MSSD is a direct measure of contour surfaces, and therefore much more sensitive to local deviations in shape, and (d) the UM95 represents the volume expansion needed to account for contouring errors. Taking this into account, the statistical results were interpreted to mean that even though the AGS tool may be sufficient in terms of centroid position and volume, it's overall shape was often incorrect. This is of great concern when considering adaptive RT, which aims to modify the beam aperture such that it conforms to the boundary of the target. Furthermore, this difference in shape manifested itself in an increase in the UM95, suggesting that AGS segmentation errors would likely have a dosimetric effect.

*Future work:* This work highlights that there remains a need for a segmentation technique that is capable of conforming to the uterine boundaries at the time of treatment to accurately represent the position and shape of the RT target. Although the AGS tool is capable of achieving this in US images with excellent image quality, it is inaccurate and unreliable in images where the uterine boundary is blurred or partially obstructed. To overcome some of the pitfalls of the AGS tool, a new algorithm is being developed that is less dependent on image gradient to semiautomatically segment the uterus; one potential solution includes incorporating shape models into a gradient‐based segmentation framework to overcome errors associated with US shadowing.^29^, ^45^ Additional work will investigate methods of improving US image quality, image processing techniques to further distinguish the uterus from surrounding tissues, quantitative methods of directly comparing other imaging modalities (such as MRI, CT, and CBCT) with US in the ability to accurately represent the uterus, and dosimetric studies assessing the relationship between uterine segmentation accuracy and target coverage and OAR sparing.^41^

## 5 Conclusions

The good agreement between manual contours when compared with results from other imaging modalities such as CT and MRI supports the use of transabdominal US to visualize the uterus prior to RT treatment for cervical cancer patients. The AGS tool was able to accurately determine the uterine shape of cervical cancer patients as well as manual contouring in cases where the image quality was excellent, but not in cases where image quality was degraded by common artifacts such as shadowing and signal attenuation. The AGS tool should be used with caution for adaptive RT purposes, as it is not reliable in accurately segmenting the uterus on ‘good’ or ‘poor’ quality images. However, there may be potential to improve the performance of the AGS algorithm if the US image quality is improved. The unreliable performance of the AGS tool highlights a continuing need for a rapid method of segmenting the uterus at treatment to obtain both uterine position and shape; this is a critical step in implementing US‐guided adaptive RT for patients with cervical cancer.

## Conflicts of interest

The data collected at the Herlev Hospital, University of Copenhagen site was part of a 3‐year PhD research project, which was granted by Elekta Inc.

## References

[1] Baker M , Jensen JA , Behrens CF. Determining inter‐fractional motion of the uterus using 3D ultrasound imaging during radiotherapy for cervical cancer. Proc SPIE 2014;9040:90400Y–90400Y‐10.

[2] Jadon R , Pembroke CA , Hanna CL , et al. A systematic review of organ motion and image‐guided strategies in external beam radiotherapy for cervical cancer. Clin Oncol 2014;26:185–196. 10.1016/j.clon.2013.11.031 24566332

[3] Chan P , Dinniwell R , Haider MA , et al. Inter‐ and intrafractional tumor and organ movement in patients with cervical cancer undergoing radiotherapy: a cinematic‐MRI point‐of‐interest study. Int J Radiat Oncol. 2008;70:1507–1515. 10.1016/j.ijrobp.2007.08.055 18164850

[4] Collen C , Engels B , Duchateau M , et al. Volumetric imaging by megavoltage computed tomography for assessment of internal organ motion during radiotherapy for cervical cancer. Int J Radiat Oncol Biol Phys. 2010;77:1590–1595. 20378265 10.1016/j.ijrobp.2009.10.021

[5] Bondar ML , Hoogeman MS , Mens JW , et al. Individualized nonadaptive and online‐adaptive intensity‐modulated ra diotherapy treatment strategies for cervical cancer patients based on pretreatment acquired variable bladder filling computed tomography scans. Int J Radiat Oncol Biol Phys 2012;83:1617–1623. 22270164 10.1016/j.ijrobp.2011.10.011

[6] Lim K , Small W , Portelance L , et al. Consensus guidelines for delineation of clinical target volume for intensity‐modulated pelvic radiotherapy for the definitive treatment of cervix cancerr. Int J Radiat Oncol Biol Phys. 2011;79:348–355. 20472347 10.1016/j.ijrobp.2009.10.075

[7] Mell LK , Kochanski JD , Roeske JC , et al. Dosimetric predictors of acute hematologic toxicity in cervical cancer patients treated with concurrent cisplatin and intensity‐modulated pelvic radiotherapy. Int J Radiat Oncol Biol Phys. 2006;66:1356–1365. 16757127 10.1016/j.ijrobp.2006.03.018

[8] Mundt AJ , Mell LK , Roeske JC. Preliminary analysis of chronic gastrointestinal toxicity in gynecology patients treated with intensity‐modulated whole pelvic radiation therapy. Int J Radiat Oncol Biol Phys. 2003;56:1354–1360. 12873680 10.1016/s0360-3016(03)00325-0

[9] Brixey C , Roeske J , Lujan A , Lumada S. Impact of intensity modulated radiotherapy on haemotolgic toxicity in women with gynecologic malignancies. Int J Radiat Oncol Biol Phys. 2002;54:1388. 12459361 10.1016/s0360-3016(02)03801-4

[10] Tan LT , Rusell S , Burgess L. Acute toxicity of chemo‐radiotherapy for cervical cancer: the Addenbrooke's experience. Clin Oncol. 2004;16:255–260. 10.1016/j.clon.2003.12.004 15214649

[11] Roeske JC , Lujan A , Rotmensch J , Waggoner SE , Yamada D , Mundt AJ. Intensity‐modulated whole pelvic radiation therapy in patients with gynecologic malig nancies. Int J Radiat Oncol Biol Phys. 2000;48:1613–1621. 11121668 10.1016/s0360-3016(00)00771-9

[12] Jaffray DA , Siewerdsen JH , Wong JW , Martinez AA. Flat‐panel conebeam computed tomography for image‐guided radiation therapy. Int J Radiat Oncol Biol Phys. 2002;53:1337–1349. 12128137 10.1016/s0360-3016(02)02884-5

[13] Fontanarosa D , van der Meer S , Bamber J , Harris E , O’Shea T , Verhaegen F. Review of ultrasound image guidance in external beam radiotherapy: I. Treatment planning and inter‐fraction motion management. Phys Med Biol. 2015;60:R77–R114. 25592664 10.1088/0031-9155/60/3/R77

[14] Taylor A , Powell MEB. An assessment of interfractional uterine and cervical motion: implications for radiotherapy target volume definition in gynaecological cancer. Radiother Oncol. 2008;88:250–257. 18538873 10.1016/j.radonc.2008.04.016

[15] Lachaine M , Falco T. Intrafractional prostate motion management with the Clarity Autoscan system. Med Phys Int. 2013;1:72–80. arXiv:arXiv:1011.1669v3.

[16] Mohan R , Zhang X , Wang H , et al. Use of deformed intensity distributions for on‐line modification of image‐guided IMRT to account for interfractional anatomic changes. Int J Radiat Oncol Biol Phys. 2005;61:1258–1266. 15752908 10.1016/j.ijrobp.2004.11.033

[17] Tuomikoski L , Collan J , Keyrilainen J , Visapaa H , Saarilahti K , Tenhunen M. Adaptive radiotherapy in muscle invasive urinary bladder cancer ‐ An effective method to reduce the irradiated bowel volume. Radiother Oncol. 2011;99:61–66. 21429607 10.1016/j.radonc.2011.02.011

[18] Ladak HM , Mao F , Wang Y , Downey DB , Steinman Da , Fenster A . Prostate boundary segmentation from 2D ultrasound images. Med Phys. 2000;27:1777–1788. 10984224 10.1118/1.1286722

[19] Qiu W , Yuan J , Kishimoto J , et al. User‐guided segmentation of preterm neonate ventricular system from 3‐D ultrasound images using convex optimization. Ultrasound Med Biol. 2015;41:542–556. 25542486 10.1016/j.ultrasmedbio.2014.09.019

[20] Elekta Ltd. , Software 4.0 User Guide. 0400 2014; 2014.

[21] Besl P , McKay N. A method for registration of 3‐D shapes. IEEE Trans Pattern Anal Mach Intell. 1992;14:239–256.

[22] Lobregt S , Viergever MA. A discrete dynamic contour model. IEEE Trans Med Imaging. 1995;14:12–24. 18215806 10.1109/42.370398

[23] Hu N , Downey DB , Fenster A , Ladak HM. Prostate surface segmentation from 3D ultrasound images. Proc IEEE Int Symp Biomed Imaging. 2002;30:1648–1659.

[24] Ghanei A , Soltanian‐Zadeh H , Ratkewicz A , Yin FF. A three‐dimensional deformable model for segmentation of human prostate from ultrasound images. Med Phys. 2001;28:2147–2153. 11695777 10.1118/1.1388221

[25] Carillo V , Cozzarini C , Perna L , et al. Contouring variability of the penile bulb on CT images: quantitative assessment using a generalized concordance index. Int J Radiat Oncol Biol Phys. 2012;84:841–846. 22401919 10.1016/j.ijrobp.2011.12.057

[26] Vinod SK , Lim K , Bell L , et al. High‐risk CTV delineation for cervix brachytherapy: application of GEC‐ESTRO guidelines in Australia and New Zealand. J Med Imaging Radiat Oncol. 2017;61:133–140. 27527506 10.1111/1754-9485.12509

[27] Thomson D , Boylan C , Liptrot T , et al. Evaluation of an automatic segmentation algorithm for definition of head and neck organs at risk. Radiat Oncol. 2014;9:173. 25086641 10.1186/1748-717X-9-173 PMC4123306

[28] Bondar ML , Hoogeman M , Schillemans W , Heijmen B. Intra‐patient semiautomated segmentation of the cervix‐uterus in CT‐images for adaptive radiotherapy of cervical cancer. Phys Med Biol. 2013;58:5317–5332. 23863718 10.1088/0031-9155/58/15/5317

[29] Freedman D , Radke RJ , Zhang T , Jeong Y , Lovelock DM , Chen GTY. Model‐based segmentation of medical imagery by matching distributions. IEEE Trans Med Imaging. 2005;24:281–292. 15754979 10.1109/tmi.2004.841228

[30] Zhang T , Chi Y , Meldolesi E , Yan D. Automatic delineation of on‐line head‐ and‐neck computed tomography images: toward on‐line adaptive radiotherapy. Int J Radiat Oncol Biol Phys. 2007;68:522–530. 17418960 10.1016/j.ijrobp.2007.01.038

[31] Garnier C , Bellanger JJ , Wu K , et al. Prostate segmentation in HIFU therapy. IEEE Trans Med Imaging. 2011;30:792–803. 21118767 10.1109/TMI.2010.2095465 PMC3095593

[32] Jackson MG , Ludmir J , Bader TJ. The accuracy of digital examination and ultrasound in the evaluation of cervical length. 1992;79:214–8. 10.3109/01443619209013646 1731287

[33] Paladini D. Sonography in obese and overweight pregnant women: clinical, medicolegal and technical issues. Ultrasound Obstet & Gynecol. 2009;33:720–729. 10.1002/uog.6393 19479683

[34] Artignan X , Smitsmans MHP , Lebesque JV , Jaffray DA , Van Her M , Bartelink H. Online ultrasound image guidance for radiotherapy of prostate cancer: impact of image acquisition on prostate displacement. Int J Radiat Oncol Biol Phys. 2004;59:595–601. 15145181 10.1016/j.ijrobp.2004.01.043

[35] Fargier‐Voiron M , Presles B , Pommier P , et al. Impact of probe pressure variability on prostate localization for ultrasound‐ based image‐guided radiotherapy. Radiother Oncol. 2014;111:132–137. 24631149 10.1016/j.radonc.2014.02.008

[36] Baker M , Cooper DT , Behrens CF. Evaluation of uterine ultrasound imaging in cervical radiotherapy; a comparison of autoscan and conventional probe. 2016. 10.1259/bjr.20160510 PMC5124817 27452268

[37] Nelms BE , Tomé WA , Robinson G , Wheeler J. Variations in the contouring of organs at risk: Test case from a patient with oropharyngeal cancer. Int J Radiat Oncol Biol Phys. 2012;82:368–378. 21123004 10.1016/j.ijrobp.2010.10.019

[38] Lorenzen EL , Taylor CW , Maraldo M , et al. Inter‐observer variation in delineation of the heart and left anterior descending coronary artery in radiotherapy for breast cancer: a multi‐centre study from Denmark and the UK. Radiother Oncol. 2013;108:254–258. 23891098 10.1016/j.radonc.2013.06.025

[39] Reed VK , Woodward WA , Zhang L , et al. Automatic segmentation of whole breast using atlas approach and deformable image registration. Int J Radiat Oncol Biol Phys. 2009;73:1493–1500. 18804333 10.1016/j.ijrobp.2008.07.001 PMC2729433

[40] Li XA , Tai A , Arthur DW , et al. Variability of target and normal structure delineation for breast cancer radiotherapy: an RTOG multi‐institutional and multiobserver study. Int J Radiat Oncol Biol Phys. 2009;73:944–951. 19215827 10.1016/j.ijrobp.2008.10.034 PMC2911777

[41] Behrens CF , Andreasen TB , Lindberg H , et al. Quantitative image quality evaluation of pelvic computed tomographybased imaging systems: a novel concept in radiotherapy. Acta oncol. 2013;52:1579–1582. 24024695 10.3109/0284186X.2013.818252

[42] Yu Y , Molloy J , Acton S. Three‐dimensional speckle reducing anisotropic diffusion. The Thrity‐Seventh Asilomar Conference on Signals, Systems & Computers, 2003. 2003;2:1260–1270.

[43] Abd‐Elmoniem KZ , Youssef ABM , Kadah YM. Real‐time speckle reduction and coherence enhancement in ultrasound imaging via nonlinear anisotropic diffusion. IEEE Trans Biomed Eng. 2002;49:997–1014. 12214889 10.1109/TBME.2002.1028423

[44] Ji S , Roberts DW , Hartov A , Paulsen KD. NIH public access. Med Image Comut Comput Asist Interv. 2009;12:795–802. 10.1007/978-3-642-04268-3 PMC2880830 20426061

[45] Cootes TF , Edwards GJ , Taylor CJ. Active appearance models. IEEE Trans Pattern Anal Mach Intell. 2001;23:681–685.

